# Antimicrobial film from poly(butylene succinate) and cymophenol as a sustainable approach to food waste reduction: antimicrobial properties and its effects on soil microorganism, brine shrimp (*Artemia salina*) and fresh strawberry

**DOI:** 10.1186/s13036-025-00565-1

**Published:** 2025-10-29

**Authors:** Benjatham Sukkaneewat, Kamonchai Cha-aim, Sirijutaratana Covavisaruch, Phisut Naknaen, Jakkid Sanetuntikul, Nawadon Petchwattana

**Affiliations:** 1 Division of Chemistry, Faculty of Science, Udon Thani Rajabhat University, Udon Thani, Thailand; 2 Department of Biotechnology and Agricultural Products, Faculty of Agricultural Product Innovation and Technology, Srinakharinwirot University, Nakhon Nayok, Thailand; 3 Department of Chemical Engineering, Faculty of Engineering, Chulalongkorn University, Bangkok, Thailand; 4 Department of Food Science and Nutrition, Faculty of Agricultural Product Innovation and Technology, Srinakharinwirot University, Nakhon Nayok, Thailand; 5 Faculty of Engineering and Technology, King Mongkut’s University of Technology North Bangkok, Rayong, Thailand; 6 Department of Chemical Engineering, Faculty of Engineering, Srinakharinwirot University, Nakhon Nayok, Thailand

**Keywords:** Environmental sustainability, Biodegradable polymers, Biological control agents, Food waste, Marine environment

## Abstract

To develop antimicrobial films for active food packaging applications, poly(butylene succinate) (PBS) was fabricated into films of varying thicknesses (50, 75 and 100 μm) and incorporated with different cymophenol concentrations (0 to 10 wt%). The results demonstrated that cymophenol functioned not only as an antimicrobial agent but also as a plasticizer, enhancing the essential properties required for food packaging films. The films effectively inhibited the growth of *Staphylococcus aureus* and *Escherichia coli* at cymophenol concentrations of 6 and 8 wt%, respectively. Both increased cymophenol content and film thickness contributed to improved mechanical properties, particularly by elevating stretchability. Release tests indicated that thicker films exhibited higher cymophenol migration, which correlated with an increased diffusion coefficient. Besides packaging film properties, the PBS/cymophenol plates, particularly at 100 μm thickness, presented an alternative function to enhance the shelf-life extension of strawberries by reducing yeast and mold growth. Soil microorganism vitality tests on sixteen isolated soil microbial strains showed that the antimicrobial films did not hinder the soil microbial population growth, confirming their potential to retain biodegradability. Additionally, the survival rates of brine shrimp after five days were significantly reduced when stored in PBS and PBS/cymophenol. Based on these findings, antimicrobial PBS/cymophenol films are proposed as sustainable, biodegradable active packaging materials that combine strong antimicrobial activity with environmental safety in terrestrial conditions and can be easily integrated into various food containers.

**Supplementary Information:**

The online version contains supplementary material available at 10.1186/s13036-025-00565-1.

## Introduction

Nowadays, the food supply chain, from production to consumption, generates significant food loss and waste (FLW), posing threats to sustainability, the economy, and food security [1, 2]. Waste occurs at various stages, from manufacturing to retail operations and households, often due to microbial spoilage and expiration [3]. Antimicrobial packaging, incorporating active compounds like essential oils, effectively delays spoilage by inhibiting microbial growth. This innovative solution is widely applied for shelf-life extension of foods, addressing FLW challenges [4, 5].

Over the past decade, there has been a growing interest in active food packaging with antimicrobial properties and its effectiveness in extending shelf life and reducing food waste. It can be applied to various food products, including bread, meats, seafood, fruits and vegetables [5, 6]. Heightened environmental awareness and the introduction of new sustainable packaging regulations have compelled researchers and plastic manufacturers to explore sustainable packaging alternatives. Consequently, bioplastics, e.g., poly(lactic acid) (PLA), polycaprolactone (PCL), poly(butylene succinate) (PBS) and poly(butylene adipate-co-terephthalate) (PBAT), have been adopted as polymer matrices for antimicrobial food packaging. This shift towards biodegradable options serves a dual purpose of enhancing environmental sustainability and minimizing food waste [5, 7, 8]. However, not all bioplastics can be biodegraded. Although their production increased to 2.4 million tons in 2024, approximately 55% of the bioplastics are biodegradable, with key biodegradable polymers including PBS, PBAT, PLA, polyhydroxyalkanoates (PHAs) and starch blends [9].

Production of antimicrobial packaging from the biodegradable plastic, i.e., PBS, appears to be an appropriate choice. PBS is a ductile bioplastic with an appropriate balance of flexibility, toughness, thermal properties and biodegradability [10]. In comparison to PLA, PHAs and thermoplastic starch, PBS offers better ductility, stretchability and processability. Moreover, PBS has a higher thermal stability (heat deflection temperature >90 °C) than PCL, which is a similar ductile bioplastic [10, 11]. Thus, PBS has garnered significant interest for producing food packaging films. However, PBS does not possess any inherent antimicrobial properties. The antimicrobial biodegradable packaging typically comprises a blend of active agents, such as an essential oil, within a biopolymer matrix. Cymophenol, derived from thyme and oregano plants, shows its potential as an active ingredient in such packaging owing to its favorable pharmacological properties and capability to be utilized for food additives [12]. The combination of the oil and the bioplastics has been reported to enhance the active functions of antimicrobial properties. The essential oil can be released from the polymer matrix in three distinct steps: diffusion of the oil molecules through the matrix, dissolution in food simulants, and subsequent migration to the polymer surface until equilibrium is achieved. These release processes delay or prevent the unwanted growth of microorganisms on food surface, thereby prolonging the food shelf life [7, 9].

However, it is noteworthy that the presence of an essential oil as an antimicrobial agent in an active food packaging may impact the biodegradation rate of the biopolymer matrix, potentially interfering with oxidation reactions and soil microbial functions involved in biodegradation [13]. Soil microorganisms are also important for soil quality and actively participate in the decomposition of organic matter [14]. They also promote plant cultivation. The disturbance of population growth of soil microorganisms by plastic residue or the modified plastics, i.e., antimicrobial polymers, may lead to reduced plant germination and growth rates [15]. Therefore, the antimicrobial effects of the active food packaging on soil microorganism vitality should be clarified.

To date, the development of antimicrobial biodegradable packaging is still of interest, especially for film products [16]. Antimicrobial biodegradable films have been widely investigated as they can be incorporated into many types of food packaging, such as wrapping films, coating materials for take-out food boxes and compostable bags, among others. However, to the best of our knowledge, although several studies have reported on the antimicrobial films, their focus seemed to be the improvement of antimicrobial properties. Some other necessary properties for the packaging film have been discussed. However, most requisite properties for potential applications have not been comprehensively discussed in a single work. These factors include static and dynamic mechanical performances, stretchability and toughness improvements, tear and impact resistance, morphological integrity, thermal transition behavior, crystallinity of the modified films, barrier properties and, especially, the vitality allowance for both soil microorganisms and small aquatic organisms. Comparison of these film properties among different studies appears inappropriate due to the dissimilar types of antimicrobial agents, polymer matrices, film thickness as well as experimental and testing conditions. Moreover, as aforementioned, there is great ambiguity about whether antimicrobial polymers disturb the soil microbial growth, which is needed for their biodegradation.

Therefore, the current work aims to comprehensively study the above-mentioned properties of PBS/cymophenol films. Furthermore, the effects of the essential oil contents as well as film thicknesses on the requisite properties of the biodegradable packaging films were also investigated. The reported findings will be beneficial for those working in related fields and will contribute useful information for the further design of active food packaging.

## Materials and methods

### Raw materials for film preparation

A bio-PBS (FZ91PM) with a 115 °C melt temperature and a 1.26 g/cm³ density was obtained from PTT MCC Biochem and utilized as a film matrix. The essential oil, cymophenol or 5-isopropyl-2-methylphenol (C_10_H_14_O) with a 98% (v/v) concentration, a 150 g/mol molecular weight, a 0.977 g/cm³ density and a 240 °C boiling point, were purchased from Sigma-Aldrich.

### Microorganisms

Strains of *Staphylococcus aureus* (*S. aureus*, TISTR 1466) and *Escherichia coli* (*E. coli*, TISTR 780) were obtained from the culture collection maintained by the Thailand Institute of Scientific and Technological Research (TISTR).

### Preparation of antimicrobial films

To prepare antimicrobial PBS/cymophenol films, PBS pellets were initially dry-mixed with the cymophenol oil at 2 to 10 wt% concentrations in a high-speed mixer (Thermo Prism Pilot 3) at 500 rpm for 30 s, ensuring the homogeneous dispersion of cymophenol particles within the PBS matrix. Then, the dry-blended compositions underwent melt-mixing in a twin-screw extruder (Chareon Tut, CTE-D22L32) under temperatures ranging from 100 to 150 °C and screw speeds of 80 to 100 rpm. After extrusion, each formulation was pelletized and then sheeted to produce the films with 50, 75 and 100 μm thicknesses using a sheet extruder equipped with chill rolls (Chareon Tut, SE-D50L30) for further testing and characterizations.

### Testing and characterizations

#### Antimicrobial activities and inhibition clear zone

The antimicrobial activities of PBS/cymophenol films were studied using an agar diffusion method, following literature protocols [7]. The sample dimensions were 10 mm × 10 mm. Film thicknesses (50, 75 and 100 μm) with varied concentrations of the active agent (0 to 10 wt%) for each thickness were evaluated for their effects on antimicrobial properties. *E. coli* (Gram-negative, TISTR 780) and *S. aureus* (Gram-positive, TISTR 1466) were selected as the target bacteria for testing. Inhibition zone diameters were measured to estimate the antimicrobial activities of the modified films.

#### Mechanical properties

Necessary mechanical properties of food packaging films, e.g., tensile properties, impact strength and tear resistance, were investigated. Neat PBS films at equal thicknesses were used as control samples. All specimens were kept at 23±2 °C and 50±5% RH for 48 h before measurement. For each study, the average of at least five replicate values were reported. The tensile properties were examined using a Universal Testing Machine (Instron 5567). Following ASTM D882, the rectangular film samples (10 mm × 100 mm) were subjected to tensile stress under a 1 kN of load cell and a crosshead speed of 12.5 mm/min. The impact resistance was evaluated in accordance with ASTM D3420 using a pendulum impact tester (Digital Impact Tester, Japan). A pendulum providing an impact energy of 1.5 J was employed to test the film specimens (100 mm × 100 mm). The tear resistance tests were conducted following ASTM D1922. The film specimens were tested in the machine stretching direction.

#### Microscopic observation

The fracture surfaces of neat PBS and PBS/cymophenol films were observed under scanning electron microscope (SEM, JSM 5410LV) with a 15 kV accelerating voltage. Before SEM observation, the films were broken in liquid N_2_ to produce brittle fracture surfaces and then coated with gold to avoid the electron charging effect.

#### Thermal properties

Thermal transition temperatures of neat PBS and PBS/cymophenol films were investigated using a differential scanning calorimeter (DSC) from TA Instruments (DSC2500) under N_2_ atmosphere. Initially, the sample was subjected to a heating process to eliminate the effects of prior thermal treatments. This process started from 30 to 150 °C using a heating rate of 10 °C/min. Once it reached 150 °C, it was isothermally maintained at this temperature for 10 min. Then, it underwent a cooling phase, decreasing from 150 to 30 °C at the same rate. A second and identical heating cycle was conducted. Finally, the samples were cooled to room temperature. Crystallization temperature (*T*_c_), melting temperature (*T*_m_) and enthalpy of melting (Δ*H*_m_) data were collected. The degree of crystallinity (*X*_c_) was calculated using Eq. (1).

1 $${X_{\text{c}}}=\frac{{\Delta {H_{\text{m}}}}}{{\Delta {H_{\text{f}}} \times {X_{{\text{PBS}}}}}} \times 100$$

where Δ*H*_m_ and *X*_PBS_ represent the melting enthalpy and mass fraction of PBS, respectively. Δ*H*_f_ signifies the heat of fusion, which is the melting enthalpy of 100% crystalline PBS. Its value was 110.3 J/g [7]. The glass transition temperature (T_g_) was evaluated from loss modulus peaks, which were obtained from the tension mode of a dynamic mechanical analyzer (DMA, NETZSCH, DMA 241) over a temperature range from −70 to 80 °C at a heating rate and frequency of 2 °C/min and 1 Hz, respectively.

#### Barrier properties

Barrier properties of the films, including the water vapor transmission rate (WVTR) and oxygen transmission rate (OTR), were investigated using a specimen area of 50 cm^2^. According to ASTM D3985, the WVTR was measured using a Mocon PERMATRAN-W 398 tester under N_2_ flow rate of 250 cm^3^/min, 37.8 °C and 90% RH. The OTR was measured under the O_2_ flow rate of 20 cm^3^/min using a Mocon OX-TRAN, 2/21, following ASTM D3985. For the OTR tests, the film samples were pre-equilibrated at 23±2°C and 50±5% RH for 48 h before testing. Average WVTR and OTR values were acquired from five replicate specimens.

#### Release of cymophenol

The release of cymophenol from PBS films was studied in distilled water. The testing procedures and conditions followed the EN 13130 − 2005 procedure. Quantitative analysis was conducted using High-Performance Liquid Chromatography (HPLC, Agilent 1100 series) on the samples that were immersed in distilled water for 2, 6, 12 and 24 h, as well as 2, 5, 10 and 15 days. The mobile phase consisted of 0.05 M ortho-phosphoric acid and acetonitrile in a 40:60 (v/v) ratio. The injection volume was 5 µL with a 1 mL/min flow rate.

To estimate the release of cymophenol in distilled water, mass transport parameters were calculated using Fick’s law of diffusion. This approach is based on the following assumptions: (i) the release process is governed by the Fickian diffusion model, (ii) cymophenol is uniformly dispersed within the PBS film, (iii) the initial cymophenol concentration in distilled water is zero, (iv) there are no interactions between the food simulants and the PBS/cymophenol film and (v) cymophenol and PBS remain chemically stable with no degradation during the process. The diffusion coefficients (*D*, m²/s) for cymophenol were determined using Eqs. (2) to (4), as described by Chung et al. [17].

2 $$\frac{{{M_{{\text{F,t}}}}}}{{{M_{{\text{F}},\infty }}}}=1 - \sum\limits_{{n=1}}^{\infty } {\frac{{2\alpha (1+\alpha)}}{{1+\alpha +{\alpha ^2}q_{{\text{n}}}^{2}}}} \exp \left[ {\frac{{ - Dq_{{\text{n}}}^{2}t}}{{L_{{\text{p}}}^{2}}}} \right]$$

3 $$\alpha =\frac{{{K_{{\text{FP}}}}{V_{\text{F}}}}}{{{V_{\text{P}}}}}$$

4 $${K_{{\text{FP}}}}=\frac{{{C_{{\text{F}},\infty }}}}{{{C_{{\text{P}},\infty }}}}$$

5 $$\tan {q_{\text{n}}}= - \alpha {q_{\text{n}}}$$

where *M*_F, t_ is the mass of cymophenol migrated into distilled water at time t. *M*_F,∞_ is the mass of cymophenol migrated into distilled water at the equilibrium stage. *L*_P_ is the PBS/cymophenol film thickness. *K*_FP_ is the partition coefficient of the cymophenol active compound between the distilled water and PBS, which can be calculated by Eq. (4). *C*_F,∞_ and *C*_P,∞_ are the concentrations of cymophenol in distilled water and in PBS at equilibrium, respectively. *V*_F_ and *V*_P_ are the volumes of distilled water and PBS, respectively. The *q*_n_ is the positive root of tan *q*_n_, which can be estimated using Eq. (5). The calculation was done by plotting f(*q*_n_) = tan *q*_n_ + α*q*_n_ as a function of *q*_n_ and then observing the points where f(*q*_n_) becomes zero [17].

Equation (2) was simplified by Chung et al. [17]. Thus, the diffusion coefficient, *D*, value can be estimated from Eq. (6).

6 $${\left[ {\frac{1}{\pi } - \frac{1}{\alpha } \cdot \frac{{{M_{{\text{F,t}}}}}}{{{M_{{\text{P,0}}}}}}} \right]^{0.5}}= - \frac{{{D^{0.5}}}}{{\alpha \cdot {L_{\text{P}}}}} \cdot {t^{0.5}}+\frac{1}{{{\pi ^{0.5}}}}$$

where *M*_P,0_ is the initial amount of migrant in the PBS. However, this equation can only be applied to *M*_F, t_/*M*_P,0_ < 0.6.

#### Shelf-life extension of strawberry

**Fig. 1:**
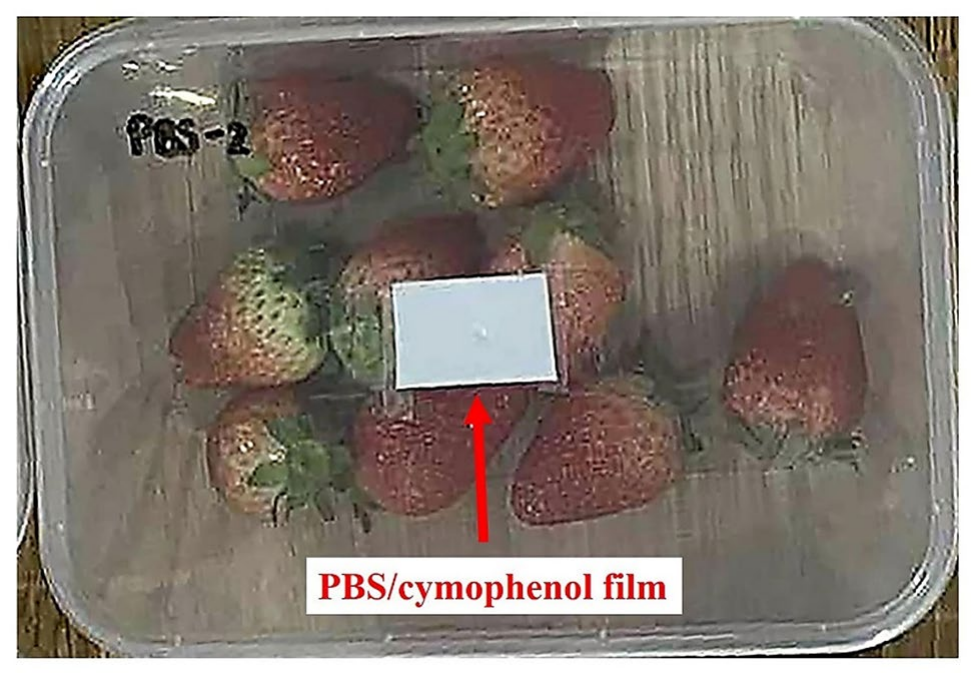
Experimental setup for shelf-life extension of strawberry

Besides the former properties for packaging film application, the effects of release efficiency of cymophenol oil on shelf-life extension of packaged strawberry were also assessed. This may expand the alternative application scopes for the PBS/cymophenol films. The experimental setup is presented in Fig. 1. The 75- and 100-μm-thick PBS/cymophenol films (50 mm × 75 mm) with 8 wt% cymophenol were incorporated with the packaging containers of strawberry. Then, the modified containers were stored in a refrigerator at 5 °C for 14 days. Total plate count of microbial growth on PCA medium at 30 °C along with yeast and mold count on YGC medium at 25 °C were performed at 0, 7 and 14 days in accordance with the FDA-BAM Chaps. 3 and 18.

#### Soil microorganism vitality

Soil microorganism vitality, affected by the antimicrobial activity of the PBS/cymophenol films, was studied for sixteen isolated soil microorganisms. The soil microbial isolation processes were conducted as follows: 1.0 g of a soil sample was soaked in 10 mL of normal saline (0.85% (w/v)) for 10 min until sediment was obtained. Then, the supernatant with soil microbial suspension was withdrawn and serially diluted. Each 100 µL diluent was cultured on nutrient agar (NA) at 30 °C for 48 h, followed by isolation of single microbial colony. Each isolated colony was further cultured at the same conditions for another 48 h. The number of soil microbial cells in the medium was determined by its measuring optical density (OD) at a wavelength of 600 nm, and the OD was adjusted in the range of 0.1 to 0.2 by dilution. Sixteen isolates of soil microorganisms were obtained. Then, the assessment of soil microorganism vitality was further conducted by detecting the accumulation capability of the microbial population over 1-month-old (the estimated cycle time of the film encompasses production, packaging, storage, logistics, shelf life, and waste management) antibacterial PBS films. Briefly, an agar diffusion method was again performed. A 100 µL aliquot of each soil microbial culture was spread onto the NA in separate Petri dishes using glass beads. Then, 75 μm-thick antimicrobial films (20 mm × 20 mm) with 8 wt% cymophenol were placed onto the dishes and incubated at 30 °C for 72 h. Either soil microbial population growth over the films or the inhibition zone was carefully detected to assess the allowance for soil microorganism vitality.

#### Vitality of brine shrimps (Artemia salina)

Saltwater was prepared by dissolving 1.8 kg of Marinium Reef sea salt in deionized water. This is a type of salt used for coral and marine fish aquaria. The mixture was stirred. Then, salinity was measured to ensure that it was 30 to 35 ppt. The composition of saltwater used in this study is presented in Table 1.

**Table 1:** Composition of saltwater from Marinium Reef sea salt

Chemical name	Chemical formula	Concentration (g/L)
Ammonium chloride	NH_4_Cl	2.00±0.05
Synthetic sea salt	-	17.5±0.05
Magnesium sulfate	MgSO_4_•7H_2_O	2.00±0.05
Potassium nitrate	KNO_3_	0.50±0.05
Potassium phosphate	K_2_HPO_4_•3H_2_O	0.10±0.05

The 1-month-old neat PBS and PBS with 8 wt% cymophenol films were cut into 50 × 50 mm squares. Each film was then placed into the synthetic seawater solution, in a 500 mL Erlenmeyer flask with no oxygen addition, together with ten brine shrimps. The brine shrimps were kindly donated by the Institute of Marine Science, Burapha University, Thailand. Their vitality was monitored through microscopic visual inspection upon exposure to the saltwater, together with pH monitoring. Three replicate samples were used for each storage condition. These were Marinium Reef saltwater (control), neat PBS in Marinium Reef saltwater and PBS/8 wt% cymophenol in Marinium Reef saltwater (200 mL). Vitality was evaluated by recording the number of surviving brine shrimps every day for a 5 days. The survival rate was calculated using Eq. (7) [18].

7 $$\eqalign{ & {\rm{Survival}}\,{\rm{rate}}\left({\rm{\% }} \right) \cr & {\rm{ = }}{{{\rm{Number}}\,{\rm{of}}\,{\rm{live}}\,{\rm{individuals}}\,{\rm{at}}\,{\rm{the}}\,{\rm{end}}\,{\rm{of}}\,{\rm{period}}} \over {{\rm{Number}}\,{\rm{of}}\,{\rm{live}}\,{\rm{individuals}}\,{\rm{at}}\,{\rm{start}}\,{\rm{of}}\,{\rm{period }}}} \times 100 \cr}$$

#### Statistical analysis

The experiment followed a completely randomized design (CRD). Data were analyzed using analysis of variance (ANOVA), and mean comparisons were conducted using Duncan’s multiple-range test.

## Results and discussion

### Antimicrobial activities

**Fig. 2:**
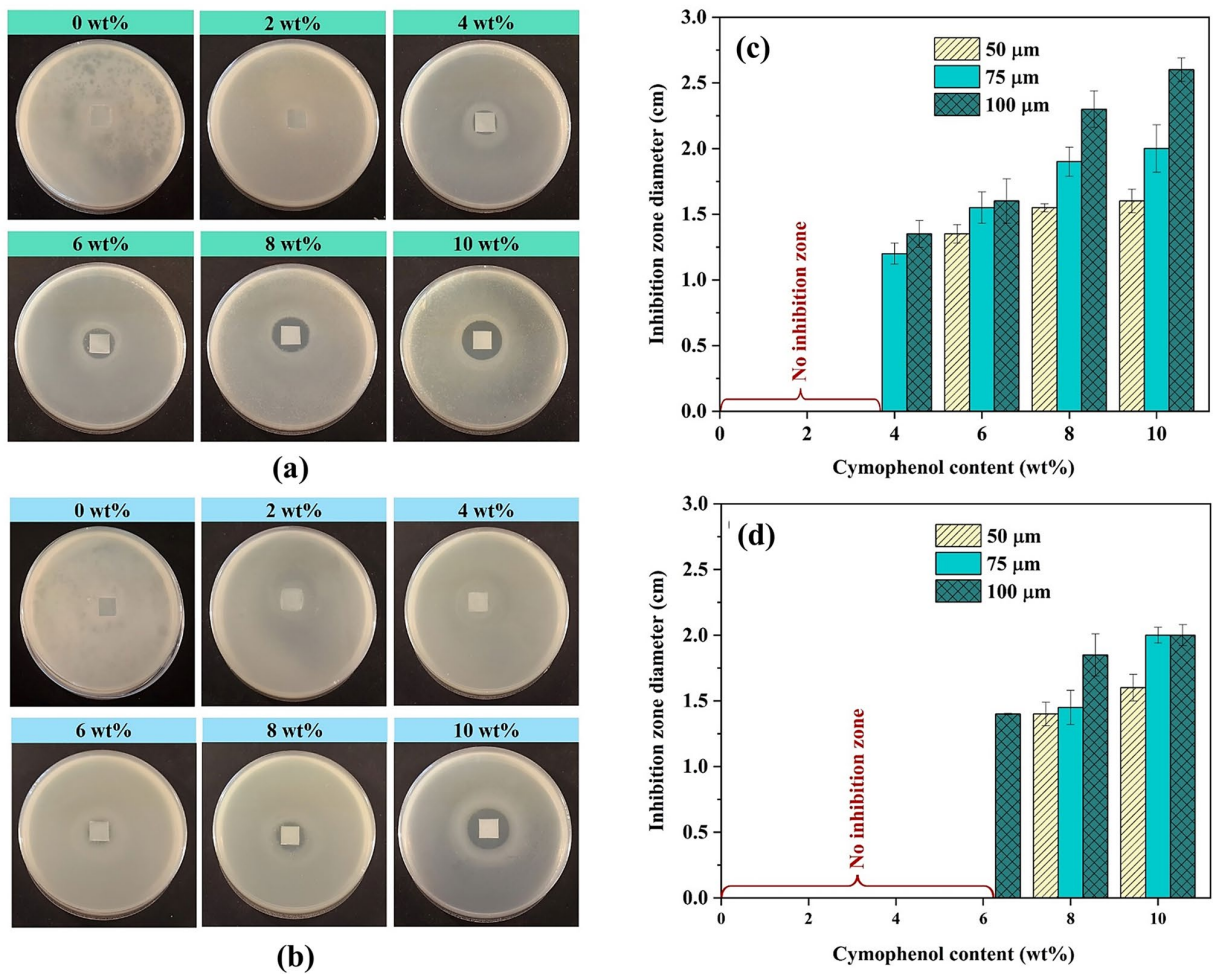
Antimicrobial activity of PBS/cymophenol films at 75 μm of film thickness and different cymophenol concentrations for (**a**) *Staphylococcus aureus* (*S. aureus)* and (**b**) *Escherichia coli (E. coli)* and their inhibition clear zone diameters for (**c**) *S. aureus* and (**d**) *E. coli*

One of the important requirements for active food packaging is an antibacterial activity. The packaging with this activity contains antimicrobial chemicals that effectively hinder bacterial growth and preserve food quality inside the package [19, 20]. Figures 2(a) and (b) show the antimicrobial activity of PBS/cymophenol films against *S. aureus* and *E. coli*, which are frequently detected in food and cause foodborne illnesses [21]. These results were obtained from 75-µm-thick films, while those of the 50- and 100-µm-thick films are given in Figs. S1 and S2 of the Supplementary Materials and were consistent with the showcase. Clear zones were used to indicate the antibacterial capability of the films. Like typical biodegradable plastics, neat PBS films with no antimicrobial agent (0 wt% cymophenol) had no clear zone for both bacterial types, indicating their inability of the unmodified film to inhibit bacterial growth. For the PBS/cymophenol composite films, although the antimicrobial agent was incorporated, the films with insufficient levels of cymophenol at 4 and 6 wt% were still unable to inhibit the accumulation of *S. aureus* and *E. coli*, respectively. However, the antibacterial performance could be improved with increased essential oil concentrations. The films with the higher levels of 6 and 8 wt% cymophenol obviously exhibited the inhibition zones against *S. aureus* and *E. coli*, respectively. These results indicated the effective concentrations of cymophenol to achieve the active function in terms of antibacterial properties for the PBS packaging films. Cymophenol served as a proton exchanger that contained hydroxyl groups and delocalized electrons for contributing to the exchange processes of protons (H^+^) inside and potassium ions (K^+^) outside the bacterial cytoplasm. This leads to cell membrane destruction, leakage of cytoplasmic ions and, eventually, cell inactivation [22].

Based on the inhibition zones in the agar diffusion tests, the active agent was more effective against *S. aureus* than *E. coli*, resulting in a larger inhibition zone for the former bacterium. The performance difference was attributed to the structural disparities between Gram-positive and Gram-negative bacterial cell walls. Gram-positive bacteria, such as *S. aureus*, primarily consist of peptidoglycan, allowing cymophenol molecules to easily penetrate and disrupt their cells. Conversely, Gram-negative bacteria, like *E. coli*, possess an outer membrane that consists of a double layer of phospholipids and lipopolysaccharides [23, 24]. This structural distinction renders Gram-negative bacteria more resistant to cymophenol than their Gram-positive counterparts [23].

Considering the effects of film thickness on antimicrobial properties, the qualitative data, specifically for the inhibition zone diameters, were evaluated as presented in Figs. 2(c) and (d). The inhibition zone diameter increased with greater film thicknesses for both *S. aureus* (Fig. 2(c)) and *E. coli* (Fig. 2(d)), indicating higher antibacterial activity for thicker films. The thicker films contained greater mass volumes of cymophenol entrapped in a PBS matrix. According to Fick’s first law of diffusion, this elevated the concentration gradient in the molecular diffusion process, which had more influence over path length [25]. The elevated concentration gradient possessed more effort to drive the cymophenol solute molecules to randomly move from an area of high concentration in the PBS matrix to an area of low concentration at the interface between the film and the agar medium. This increased the amount of active ingredient that diffused around the film to hinder the bacterial growth. Therefore, a larger inhibition zone diameter of the thicker film could be observed.

It is interesting to note that a different result was reported in other work. The study has demonstrated that the release of essential oil was inversely proportional to the film thickness. The thinner film tended to release essential oil more rapidly than the thicker one [26]. This finding shows that, aside from diffusion phenomenon, another factor may influence the retention and release of the essential oil from the antimicrobial film. To clarify, attention was paid to the interaction between the essential oil and the film. Several studies reported that improved intermolecular interaction between the film components increased the essential oil retention and enhanced the antibacterial properties. For example, Zhang et al. [27] thickened the film using a multi-layer technique to improve the interaction between the polymer film and cinnamon oil, while, Xu et al. [28] adjusted the blending ratio of the polymer film to elevate the electrostatic interactions to contain greater amounts of the same oil. As a result, they improved both the oil retention and the antimicrobial performance of the modified films. About this work, the cymophenol has a hydroxyl group that can interact with the oxygen atom of PBS to form hydrogen bonds. This intermolecular interaction, which was augmented by increased film thickness, could help to retain the cymophenol molecules, retard the diffusion rate and, subsequently, promote slow-release properties. In addition to the concentration gradient, this also led to the ameliorated antimicrobial properties of the thicker film. However, an improved antimicrobial result seems a weak justification. More data, especially on the film’s mechanical properties, is needed to understand this observation. For instance, if the intermolecular interaction between the cymophenol and PBS can be improved as expected, the mechanical properties of the composite film would be enhanced as well.

Fortunately, the dissimilar findings among the research studies are useful, especially for packaging design. They indicate how the choice of film thickness in packaging applications can impact the shelf life of packed products. Thinner films with rapidly released essential oils were found to be more effective in extending the shelf life of food products, while the thicker films might offer slower release rates, which could be advantageous for longer-term preservation. Therefore, thinner films may be preferred for short-term preservation, while thicker films may be suitable for long-term storage.

### Mechanical properties

The potential applications of PBS films in several types of food packaging, e.g., wrapping films, coating materials for take-out food boxes, and compostable bags, among other applications, require films with sufficient flexibility and toughness to reach their desirable stretchability. Whereas, impact and tear resistance are needed to prevent damage to the packaging films during their transport and storage at low temperatures [16].

**Fig. 3:**
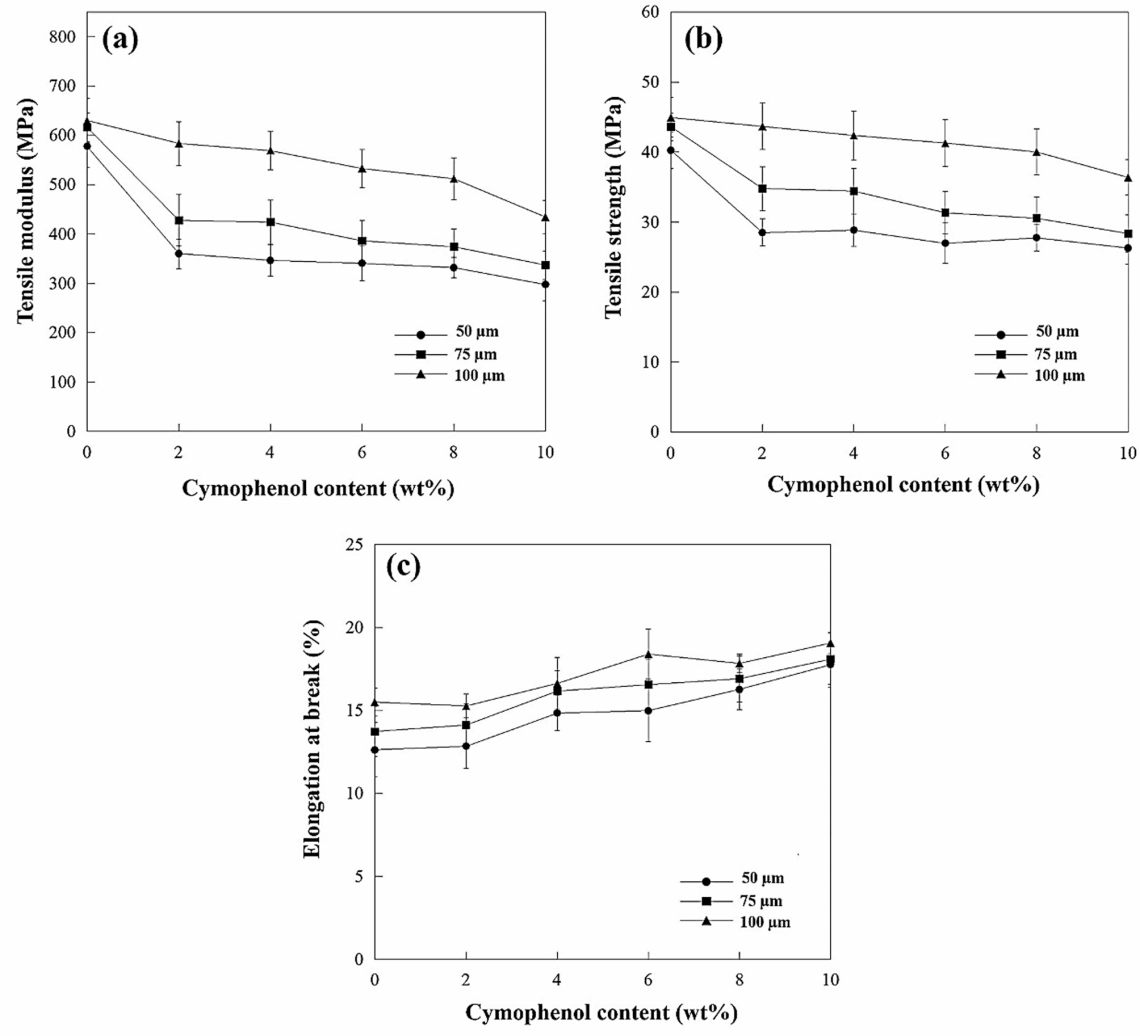
Tensile properties of the PBS films at different cymophenol contents and film thicknesses; (**a**) tensile modulus, (**b**) tensile strength and (**c**) elongation at break

To investigate such mechanical properties, the static tests (for tensile performance and tear resistance) and dynamic test (for impact resistance), affected by various cymophenol concentrations and film thicknesses, were examined for the PBS/cymophenol films. Figures 3(a) and (b) show that addition of cymophenol to PBS resulted in a noticeable decrease in both the tensile modulus and strength, which decreased approximately 10 to 40%, depending on the cymophenol content. These results indicated that the added cymophenol oil provided more flexibility to the modified PBS films. In comparison to neat PBS, these blended films exhibited reduced rigidity, primarily due to the plasticization effect imparted by cymophenol oil. As a result, the PBS/cymophenol blends displayed improved stretchability and deformability, compared to their unmodified counterparts. Film thickness slightly affected the modulus and strength. The 100 μm thickest film was found to have the highest tensile modulus and strength owing to its densest molecular chain packing, which required more force for deformation than the thinner films.

**Fig. 4:**
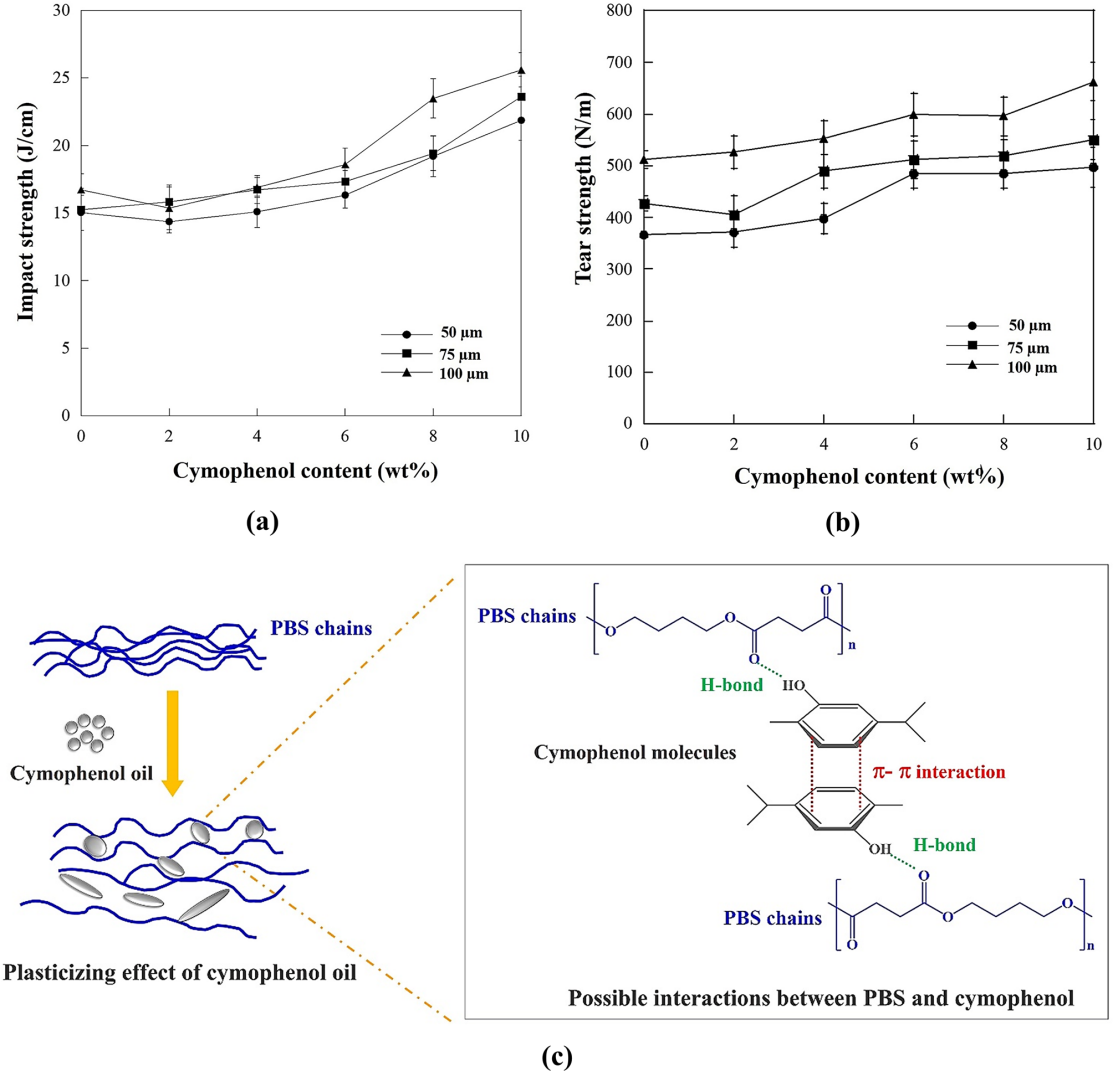
Properties of PBS films at various cymophenol contents and film thicknesses; (**a**) impact strength, (**b**) tear strength and (**c**) a proposed molecular model of the plasticizing mechanism and possible interactions between PBS and cymophenol molecules

Toughness is a crucial mechanical property of these films. Packaging films that lack suitable toughness cannot be used in practical applications [29, 30]. The improved toughness of film materials is shown by an increased elongation at break, impact resistance or tear strength [31]. As observed, the tensile elongation at break in Fig. 3(c) and the impact strength in Fig. 4(a) significantly increased with higher cymophenol contents. At the equal cymophenol content, both elongation at break and impact strength slightly increased with greater film thicknesses. PBS is not an excessively brittle polymer and can deform a bit under loading [11, 20]. Thus, a greater PBS ratio in the film achieved by increasing the film thickness could slightly extend the elongation at break and increase the impact strength. At higher cymophenol oil contents, significant improvement in both elongation at break and impact strength indicated that the cymophenol molecules could penetrate through the intermolecular free volume among PBS chains. They acted as plasticizers to separate the polymer chains. This increased the molecular free volume and gave PBS chains more capability to better respond to tensile forces or absorb the impact stress. Furthermore, the oily nature of cymophenol might provide a lubricating effect that delayed specimen breakdown and promoted ductility under impact loading [32]. Figure 4(b) further illustrates the influence of film thicknesses and cymophenol concentrations on the tear resistance of the antimicrobial films. It was evident that the increases of both film thickness and cymophenol loading improved the tear strength of these antimicrobial films. This was primarily attributed to increased toughness, which likely played a role in delaying and preventing crack propagation within the films.

The plasticizing effects of the cymophenol oil in the PBS blended films aligned with previous findings about plasticization improvements in other packaging films using different essential oils. For instance, Zhao et al. [33] introduced *Flos Sophora Immaturus* flavonoid extracts into gelatin/chitosan films, which resulted in a notable increase in tensile elongation at break by more than 2000%. The plasticization effect of the essential oils was also evident in PLA films when thymol [34] and *Liquidambar Orientalis* oils [35] were incorporated. This resulted in decreased stiffness and greater toughness. However, a different finding was found in the case of a non-biodegradable film. Carvacrol and its complexes were added to low-density polyethylene (LDPE) [36]. Even though the modified LDPE films achieved the desirable antifungal activity with the inclusion of carvacrol oil, their strength and elongation at break deteriorated. This suggested that, in some cases, incorporation of an essential oil into polymers might cause reduced toughness. Reduced mechanical properties might be related to inadequate interaction between the essential oil and LDPE, since this polymer lacks functional groups for creating strong interactions with other compounds.

Herein, the increased toughness of PBS/cymophenol films in terms of elongation percentage, impact and tear strength values, together with improved antimicrobial properties, suggests potential interactions between cymophenol and the PBS matrix. Figure 4(c) presents a molecular visualization demonstrating possible interactions between cymophenol and PBS. When cymophenol molecules act as plasticizers and relocate between PBS chains, they can use their hydroxyl groups to induce hydrogen bonding with ester functional groups along the PBS chains, while still retaining the π-π interactions around their aromatic rings. These enhance intermolecular interactions between the polymer matrix and the antimicrobial agent, leading to toughness improvement for both static and dynamic mechanical properties.

Based on the antimicrobial and mechanical investigations, cymophenol oil acted not only as an antimicrobial agent but also as an processing oil to enhance the flexibility and toughness of the modified PBS films. Increasing both cymophenol concentrations and film thickness improved the toughness of the modified PBS films, but this effect was more predominant for the oil concentrations. This was related to the plasticizing effects and intermolecular interactions between the PBS matrix and cymophenol. The obtained PBS/cymophenol blends possessed the desirable features of biodegradable films for packaging applications as they showed more stretchability, while retaining higher toughness to resist crack propagation from both impact and tear stresses.

### Morphological properties

**Fig. 5:**
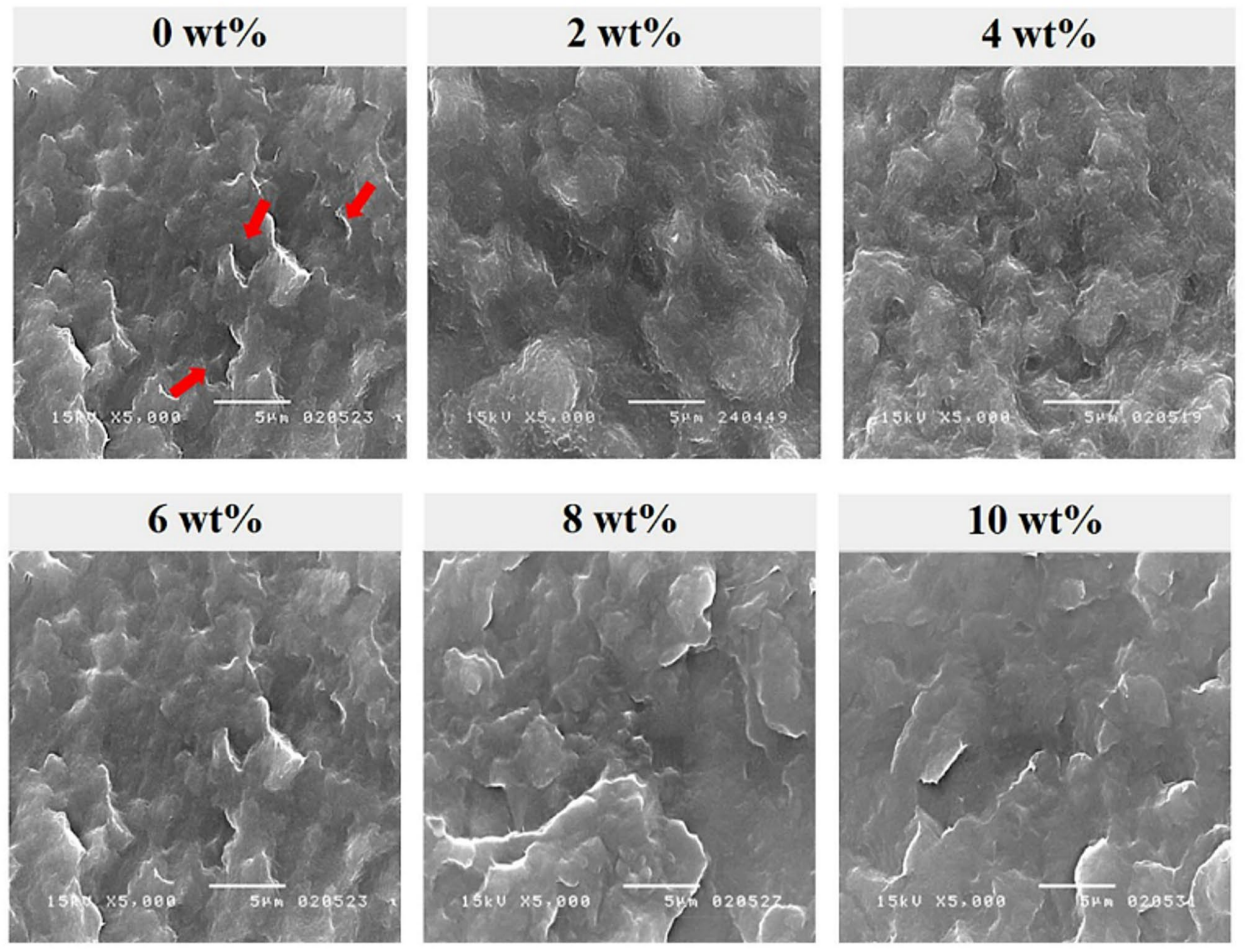
SEM microstructure of the fracture surfaces of PBS films at different cymophenol contents

Figure 5 represents a brittle fracture surface of the antimicrobial PBS films with different cymophenol contents. A neat PBS film with 0 wt% cymophenol exhibited the roughest surface with several PBS clumps owing to the aggregation of non-homogeneously melted PBS with no processing aid. The presence of PBS clumps created adjacent micro-voids, as indicated by the arrows, and decreased the microstructural integrity in the polymer matrix. The cymophenol-modified PBS films possessed smoother surfaces, especially for the formulations with 8 and 10 wt% cymophenol levels. The number and size of PBS clumps was lessened with increased cymophenol concentrations, suggesting higher homogeneity of the film surface and compatibility between the components. Wongphan et al. [37] suggested that the plasticizing effects and the compatibility of essential oil helped to improve the melting and unfolding behavior of PLA and PBAT during their processing. Also, Wan et al. [38] reported that incorporating a miscible additive in liquid form increased the polymeric chain orientation in the machine stretching direction. According to these suggestions, cymophenol, which could have the intermolecular interactions with PBS, likely served as a processing oil to facilitate the disaggregation of PBS clump particles during the melt-extrusion process. This helped to minimize the micro-voids and fine-tune the microstructural integrity of the final film product. It also enhanced the films’ mechanical properties, e.g., flexibility and ductility, owing to the morphological-mechanical relationship.

### Thermal properties

**Fig. 6:**
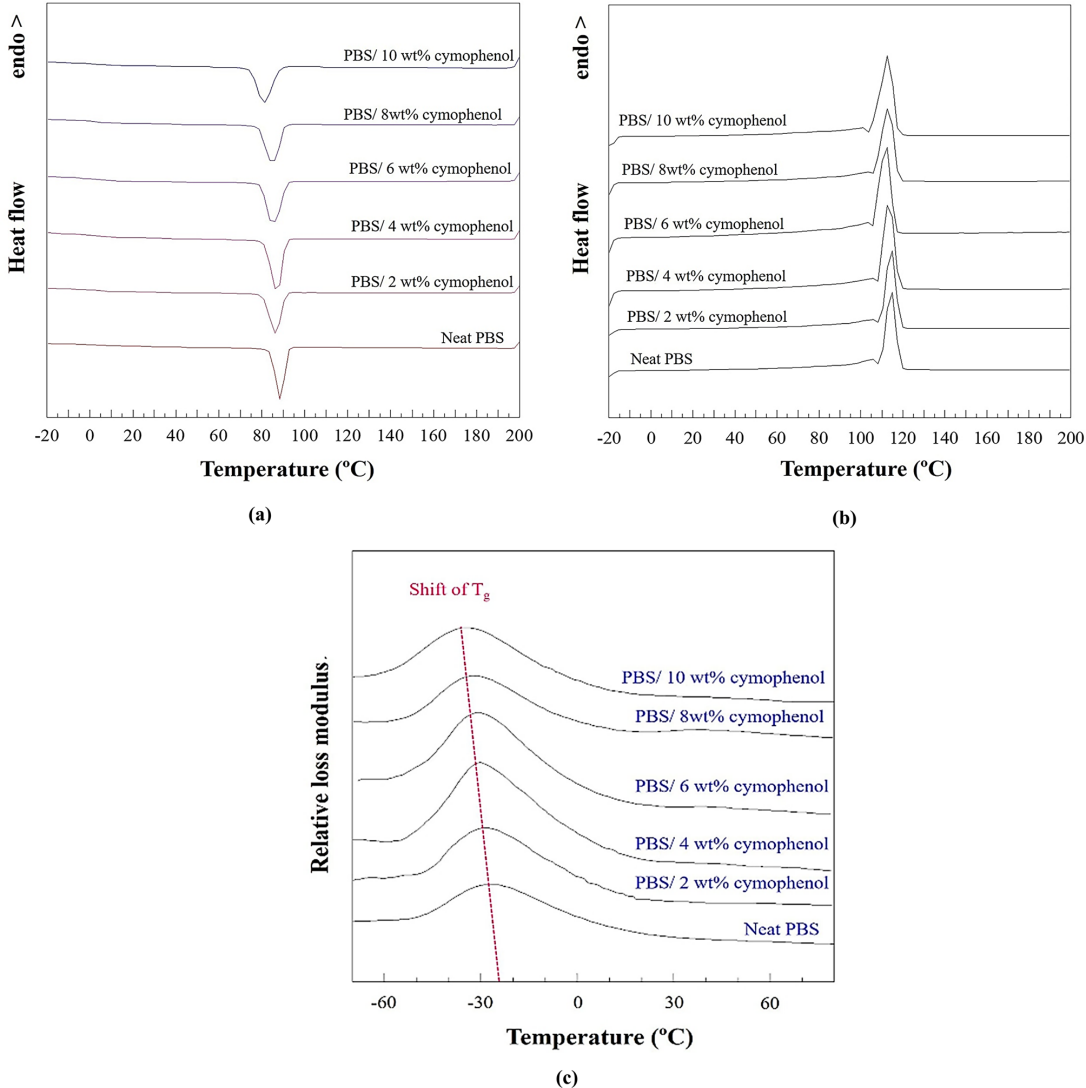
Thermal properties of the PBS films at various cymophenol contents; (**a**) the second heating cycle of DSC thermograms, (**b**) DSC cooling curves and (**c**) T_g_ shift in loss modulus graphs of DMA

Table 2 presents thermal transition parameters of neat PBS and PBS/cymophenol blends, including T_c_, T_m_, ΔH_m_ and X_c_. The DSC thermograms used to evaluate these parameters are given in Figs. 6(a) and (b). The T_c_ and T_m_ values in the DSC thermograms were shifted. In Table 2, incorporation of cymophenol into PBS resulted in a decreased T_c_ compared to neat PBS. The lowest T_c_ value, 81.0 °C, was observed when 10 wt% cymophenol was added, confirming the plasticizing effect that facilitated the increased mobility of the PBS chains [39]. Since the T_c_ value is related to the crystalline region, the reduced T_c_ implies the ease of crystalline nucleation in the cymophenol-modified PBS films [40]. T_m_ also exhibited a slight shift to an approximately 2 °C lower temperature. This is related to the gradual decrease in the ΔH_m_ from 71.1 to 51.4 J/g, which was attributed to the expansion of the amorphous region within the blends, a phenomenon supported by the decreased X_c_.

**Table 2:** Thermal properties of PBS/cymophenol films obtained from a DSC technique

Cymophenol content (wt%)	T_c_ (°C)	T_m_ (°C)	∆H_m_ (J/g)	X_c_ (%)
0	88.8	114.4	71.1	64.42
2	86.4	114.2	59.9	55.41
4	87.2	113.5	57.9	54.64
6	84.9	113.1	55.2	53.24
8	84.9	112.6	52.1	51.35
10	81.0	112.4	51.4	51.80

Additionally, the change in the glass transition temperature (T_g_) was also determined by the shift of the loss modulus peaks in DMA curves, as illustrated in Fig. 6(c). The appearance of T_g_ represents the molecular motions in the amorphous region of the polymer [40]. Films with high T_g_ values have little chain mobility and become rigid films that may not be appropriate for stretching or wrapping in food packaging applications [29, 30]. The DMA results in Fig. 6(c) suggest that the loss modulus peaks continually shifted to lower temperatures with increased cymophenol concentrations, highlighting the T_g_ reduction of the modified films. The modified film with the highest cymophenol concentration had the lowest T_g_ value, -37 °C, which decreased from that of the neat PBS film (-27 °C) by 37.0%. An explanation is that a lower T_g_ implied more chain mobility and provided higher flexibility to polymer films [40]. Several works attempted to reduce T_g_ values of polymer films using various techniques, including the use of plasticizers [41], blends with more ductile polymers [40] and tailoring the molecular architecture [42]. In this case, the capability of cymophenol to shift the T_g_ of the modified PBS film to lower values is beneficial for the applications of frozen food packaging since the packaging films can potentially retain their ductile properties in a rubbery state, even though the freezing temperature is very low.

### Water vapor and oxygen transmission rates

**Fig. 7:**
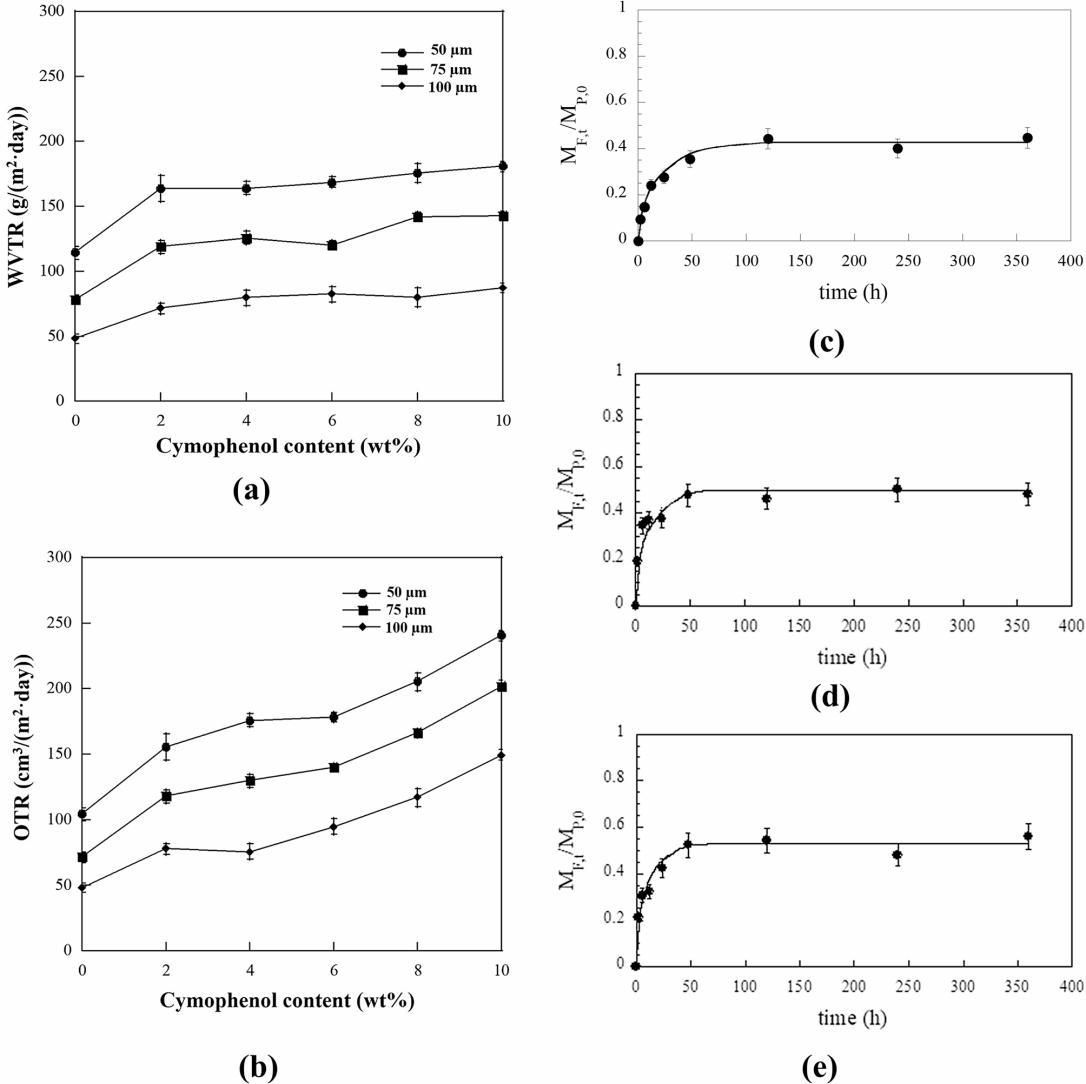
Mass transfer properties of the films; (**a**) WVTR and (**b**) OTR values and the release of cymophenol from PBS films into distilled water for (**c**) 50, (**d**) 75 and (**e**) 100 μm films

Barrier properties were investigated as they serve to protect and minimize food contamination [43]. The cymophenol concentration and the film thickness affected the barrier properties in opposite directions. The WVTR (Fig. 7(a)) and the OTR (Fig. 7(b)) decreased with greater film thicknesses, while they increased with higher cymophenol concentrations. In the permeation process, the transport of gas and vapor through polymer films generally comprises four steps, involving the permeant absorption at a film surface, the dissolving of the molecules, the diffusion of these molecules, and the desorption of the permeable species [20]. As such, the plasticizing effects of cymophenol, which elevated the amorphous segments and the intermolecular free volume, facilitated the dissolving and the diffusion steps of oxygen and water molecules through the PBS films, resulting in larger permeability. This phenomenon was in line with the findings from Ramos et al. [44]. They found the increased OTR of the antimicrobial polypropylene (PP) films with greater concentrations of thymol and carvacrol oil. Similar to the results for plasticized starch, the oxygen permeability increased with erythritol, sorbitol, and lactitol concentrations [45]. The explanation from both works was believed in the effective plasticizing effects of the plasticizer-like additives.

Although the WVTR and the OTR increased at higher cymophenol contents, they could be significantly improved at greater film thicknesses for every cymophenol concentration. For instance, at 10 wt% cymophenol (the highest level), an increased film thickness from 50 to 100 μm reduced the WVTR and the OTR to 87.2 g/(m^2^·day) and 149.4 cm^3^/(m^2^·day), respectively. These transmission rates decreased approximately 52% for WVTR and 38% for OTR, compared to the thinnest film. Generally, PBS is a semi-crystalline polymer, containing both amorphous and crystalline regions. An increased PBS film thickness also enlarged the stacked crystalline region, which blocked diffusion pathways and obstructed the permeability of small molecules like oxygen and water [20]. The WVTR and OTR results clearly show how film thickness is important and should be considered in essential oil-modified biodegradable films. In this case, although better toughness, flexibility, surface integrity and the active functions, i.e., antimicrobial activity, could be achieved by increasing the oil levels, the barrier performance could be improved by adjusting the film thickness.

To achieve the possible utilization of modified PBS/cymophenol films, potential applications as packaging films were explored. Wang et al. [46] suggested that the suitable OTR values of the films for packaged fresh meat are 30 ~ 80 cm^3^/(m^2^·day), while, those for bakery products should be in the range of 30 ~ 4,000 cm^3^/(m^2^·day). In the current study, the OTR values of the cymophenol-modified films were approximately 65–240 cm^3^/(m^2^·day). According to this suggestion, the antimicrobial PBS films in this work have the potential to be further developed as active packaging films for fresh meat and bakery products.

### Release of cymophenol

The fractional release (*M*_F, t_/*M*_P,0_) versus time curves, shown in Figs. 7(c)-(e), were generated by fitting Eq. (2) to the experimental data. This illustrates the migration of cymophenol from PBS films into distilled water. The model predictions aligned well with the experimental results, indicating that the release kinetics are accurately described by Fick’s law of diffusion. The results revealed that cymophenol rapidly migrated from the PBS films into the distilled water as incubation time increased. An equilibrium was reached within approximately 50–60 h for all film thicknesses studied. Film thickness was identified as a key factor influencing the migration rate. At thermodynamic equilibrium, the 100 μm thick film facilitated the largest fractional release of cymophenol, approximately 0.5. The diffusivity (*D*), in Table 3, shows that the diffusion process for different film thicknesses ranged from 2.45 × 10^− 16^ to 1.77 × 10^− 15^ m^2^/s. A maximal *D* value was found for the 100 μm film, likely due to the higher amount of cymophenol per film volume.

**Table 3:** Diffusion coefficients for the release of cymophenol with various film thicknesses

Film thickness (µm)	Diffusivity (m^2^/s)
50	2.45 × 10^− 16^
75	7.73 × 10^− 16^
100	1.77 × 10^− 15^

Cymophenol release from the PBS matrix occurred in three stages, similar to the other systems discussed earlier. First, cymophenol molecules penetrated the PBS matrix. Next, cymophenol molecules dissolved in the simulants, leading to mass transfer toward the PBS surface. Finally, the dissolved cymophenol migrated completely into the food simulants until thermodynamic equilibrium was achieved. This release was enhanced by the plasticization effect of cymophenol, as evidenced by an increased tensile elongation at break and a decreased ΔH_m_ [24, 44].

### Shelf-life extension of strawberry

**Fig. 8:**
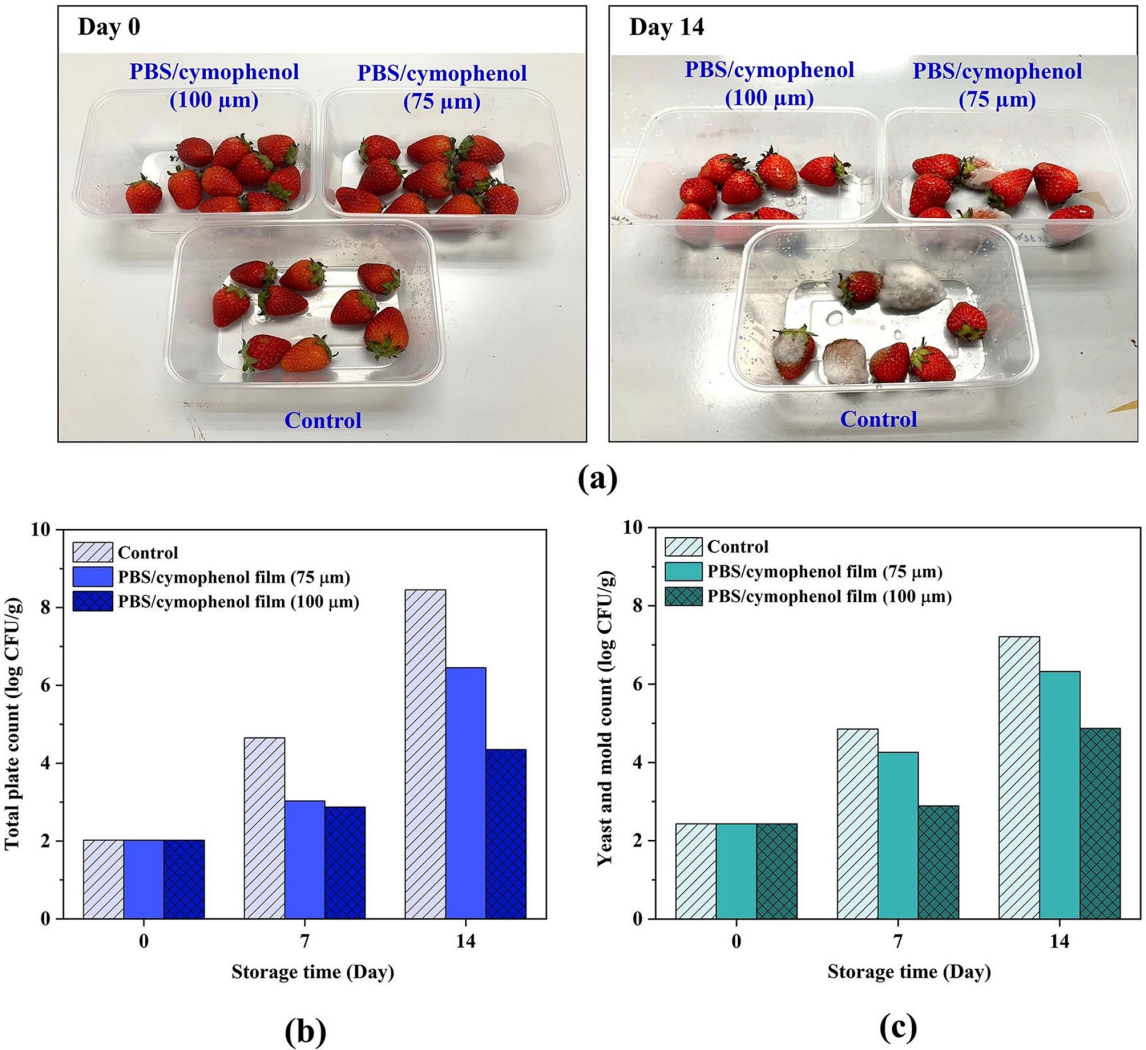
Shelf-life studies of strawberry; (**a**) appearance of strawberries with and without PBS/cymophenol films at days 0 and 14, (**b**) total plate count and (**c**) yeast and mold count

Apart from the packaging film properties, Fig. 8 shows the release capability of cymophenol oil from PBS films that affected shelf-life extension of packed strawberry. In Fig. 8(a), control strawberry samples with no antibacterial film were spoiled faster after storage for 14 days. Several strawberries became moldy with obviously increased growth of fungus. This rapid fungal growth emphasized the vulnerability of fresh strawberry to microbial spoilage under high-moisture and sugar-rich conditions, particularly in the absence of preservation mechanisms. When the PSB/cymophenol film at 75 μm thickness was applied, the strawberries showed notably suppression in fungal growth. However, trace amounts of fungal accumulation were slightly found. No visible fungal growth was observed in the case of strawberries contained with 100-µm-thick PBS/cymophenol film for all 14 days of test period. Thus, the application of PBS/cymophenol antimicrobial film signified an improvement in fungal control.

Considering a total plate count (Fig. 8(b)**)** and a yeast and mold count (Fig. 8(c)), both parameters increased over the storage time, primarily due to microbial growth under favorable conditions. The strawberries without PBS/cymophenol film exhibited the most rapid increase in the total plate count and yeast and mold count, resulting in the highest levels of both parameters at days 7 and 14. These increases in total plate count and yeast and mold count were slower when the PBS/cymophenol films were used, particularly with increased film thicknesses. Strawberry samples packaged with the thickest PBS/cymophenol film (100 μm) showed the lowest values of total plate count and yeast and mold count at both detection intervals. In comparison to the control at 14 days of storage, strawberries packed in PBS/cymophenol film (100 μm) displayed a decrease in total plate count and yeast and mold count of about 4 and 2 log CFU/g, respectively, while strawberries stored in PBS/cymophenol film (75 μm) displayed a decrease in total plate count and yeast and mold count of about 2 and 1 log CFU/g, respectively. These results again highlighted the important role of film thicknesses in regulating the cymophenol release profile. Cymophenol, a monoterpene phenol with antifungal and antibacterial properties [12], was gradually released from the PBS matrix and inhibited the microbial colonization on fruit surface. Consistent with the previous releasing results, a thicker active film with higher concertation gradient of oil facilitated greater fractional release and diffusivity of cymophenol. Therefore, the 100-µm-thick film steadily released and maintained higher local concentration of cymophenol that interfered the growth environment for spoilage organisms, such as damaging microbial cell membranes or disrupting their metabolic pathways [5]. This higher antimicrobial action of the thicker PBS/cymophenol film provided enhanced protection against both yeast and mold growth and, therefore prolonged the shelf-life of strawberries. Therefore, besides the active film for packaging applications, this approach provides an alternative function of the PBS/cymophenol films as antimicrobial plates for shelf-life extension, which can contribute a sustainable reduction of food waste.

### Vitality of soil microorganisms and brine shrimp (*Artemia salina*)

**Fig. 9:**
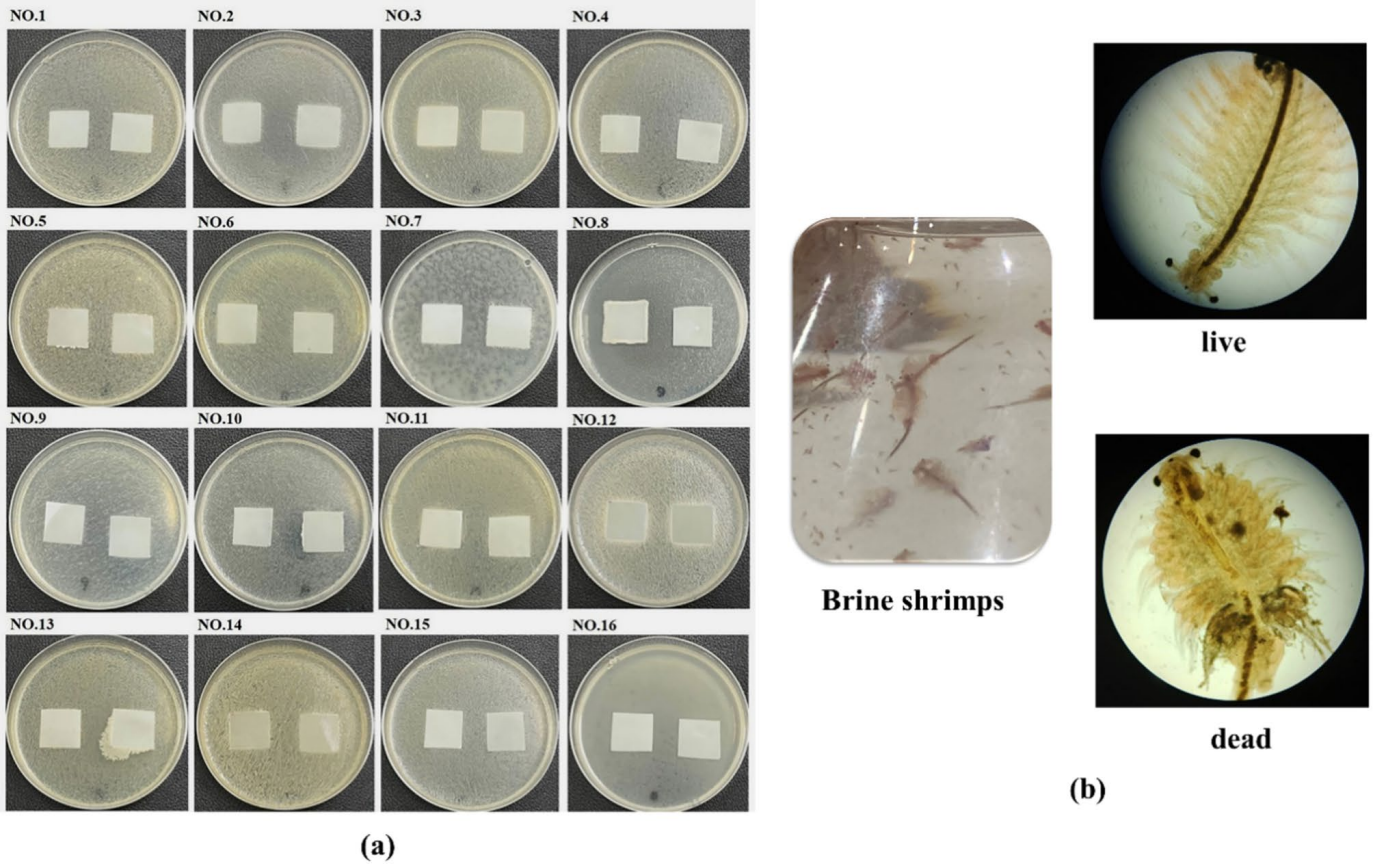
Effects of PBS/cymophenol films (8 wt% essential oil and 75 μm film thinkness) on soil microorganisms and small aquatic organisms; (**a**) vitality tests for sixteen isolated soil microorganisms and (**b**) appearance of live and dead brine shrimp

Soil microbial growth is still needed to accelerate the biodegradation processes of polymers [9, 47]. It comes to an interesting ambiguity that is whether incorporation of a cymophenol active agent in the antibacterial film may disturb the soil microorganism functions for degrading the polymer chains. Thus, the soil microbial vitality for the 75-µm-thickness films with 8 wt% cymophenol was tested against sixteen isolated soil microorganisms. These results are illustrated in Fig. 9(a). After incubation, the microbial population still increased and grew throughout the modified films. No inhibition zones were detected for any of the isolated soil microorganisms. This suggests that incorporating cymophenol oil into PBS films did not affect soil microbial vitality. Hernández-García et al. [9] and Cano et al. [13] recommended that only specific active agents derived from a metal, i.e., silver nanoparticles, significantly disturbed the ecology of soil microorganisms and delayed plastic biodegradability [9, 13].

Based on soil microorganism vitality results, the biodegradable process accelerated by the soil microorganisms, including hydrolysis of polymer chains to form tiny water-soluble fragments and microorganism assimilation into CO_2_, H_2_O and biomass [48], which led to the decomposition of the polymer matrix might be promising. However, further studies seem to be necessary to specify the actual degradation mechanisms, which may undergo either chemical reactions or enzymatic processes.

It is established that the biodegradation of PBS begins with hydrolysis. After use, PBS may potentially affect water quality and small aquatic organisms. Table 4 presents the survival rate of brine shrimp, along with pH and temperature changes over five days of storage. The vitality of the shrimp in the saltwater containing neat PBS and PBS with 8 wt% cymophenol was assessed by counting the number of live and dead individuals under a microscope **(**Fig. 9(b)). The vitality results were compared to those obtained from the control saltwater without the addition of any PBS or PBS/cymophenol films. 

On day 5, the survival rates were 63.3, 43.3, and 36.7% for shrimp stored in saltwater (control), neat PBS in saltwater, and PBS with 8 wt% cymophenol in saltwater, respectively. In this case, a reduced pH, resulting from the release of succinic acid, dimethyl ester, glutaric acid, dimethyl ester and adipic acid [49], likely impacted saltwater quality. Additionally, the presence of an oil-like substance such as cymophenol might hinder oxygen diffusion into the saltwater, which further reduced the survival rate of the brine shrimp. These findings aligned with the previous studies, indicating that pH stability was crucial for the viability of aquatic organisms [50, 51]. Further research is needed to explore the long-term effects of cymophenol on brine shrimp survival and its potential applications in marine organism preservation.

Based on these results, it can be initially concluded that PBS and PBS/cymophenol films seem not suitable for marine use. However, since the experiment was conducted at small scale, the effects of PBS and PBS/cymophenol films on the storage conditions may have been amplified.

**Table 4:** Survival of brine shrimp (*Artemia salina*)

Storage condition	Tests	Monitoring period				
		Day 1	Day 2	Day 3	Day 4	Day 5
Marinium Reef saltwater (control)	Survival rate (%)	100±0.00^**aA**^	96.7±5.77^**aA**^	80.0±10.0^**aBC**^	63.3±5.77^**aD**^	63.3±5.77^**aD**^
	pH	8.81±0.04	8.77±0.04	8.78±0.03	8.72±0.04	8.75±0.06
	Temperature (°C)	30.7±0.58	30.3±0.5	30.3±0.58	31.3±0.58	31.3±0.58
Neat PBS in Marinium Reef saltwater	Survival rate (%)	100±0.00^**aA**^	83.3±5.77^**bB**^	66.7±5.77^**aC**^	50.0±0.00^**aDE**^	43.3±5.77^**bB**^
	pH	8.80±0.05	8.73±0.06	8.67±0.08	8.56±0.07	8.53±0.12
	Temperature (°C)	30.7±0.58	31.3±0.58	30.7±0.58	31.0±0.00	30.7±1.15
PBS/8 wt% cymophenol in Marinium Reef saltwater	Survival rate (%)	100±0.00^**aA**^	86.7±5.77^**abAB**^	76.7±5.77^**aBC**^	56.7±15.3^**aD**^	36.7±5.77^**bB**^
	pH	8.81±0.09	8.76±0.12	8.71±0.05	8.70±0.05	8.71±0.05
	Temperature (°C)	30.7±0.58	31.3±0.58	30.3±0.58	31.3±0.58	31.0±1.00

Lowercase letters indicate significant differences between columns

Uppercase letters indicate significant differences between row

## Conclusions

In this study, PBS/cymophenol films were successfully prepared. The results indicated that cymophenol probably had intermolecular interactions with PBS, functioning as an effective plasticizer. These also enhanced a well-integrated morphology of the films. Consequently, the modified films exhibited improved flexibility and toughness, as reflected by increased elongation, impact resistance and tear strength at higher cymophenol contents. Although greater film thickness also contributed to these enhancements, its effect was comparatively minor. DSC and DMA analyses revealed that PBS/cymophenol films had lower glass transition and crystallization temperatures, along with decreased melting enthalpy and crystallinity, compared to neat PBS. This suggests an expanded amorphous region within PBS matrix, and consequently, resulted in the increase of water vapor and oxygen transmission rates. Nonetheless, both barrier properties could be significantly improved by increased film thickness. Regarding its active functionality, PBS/cymophenol films demonstrated antimicrobial activity against *S. aureus* and *E. coli* at 6 and 8 wt%, respectively. Increased film thickness further enhanced this antimicrobial effect by promoting concentration gradient. Cymophenol rapidly migrated from PBS films into water, reaching equilibrium within 50 to 60 h. The highest diffusion value was observed for the 100-µm-thick film. In shelf-life extension study, the thicker PBS/cymophenol films effectively delayed microbial spoilage of strawberries by reducing total plate and fungal counts. Despite the antimicrobial properties, the PBS/cymophenol films still supported the accumulation and vitality of soil microorganisms, suggesting the retention in their biodegradability potential. In small-scale marine environments, the survival rates of brine shrimp after five-day storage in saltwater (control), neat PBS in saltwater, and PBS/cymophenol (8 wt%) in saltwater were 63.3, 43.3, and 36.7%, respectively.

Based on their properties, the antimicrobial PBS/cymophenol films show the potential as the active biodegradable food packaging and offer the alternative approach for shelf-life extension and food waste minimization. However, further studies are required to confirm marine applicability under the larger-scale experimental conditions.

## Supplementary Information

Below is the link to the electronic supplementary material.

Supplementary Material 1

## Data Availability

No datasets were generated or analysed during the current study.
