# Supplementary material for: Antimicrobial film from poly(butylene succinate) and cymophenol as a sustainable approach to food waste reduction: antimicrobial properties and its effects on soil microorganism, brine shrimp (Artemia salina) and fresh strawberry
Source: J Biol Eng. 2025 Oct 29;19:92. doi: 10.1186/s13036-025-00565-1 (PMC12573842; doi:10.1186/s13036-025-00565-1)
Supplement: Supplementary file 1 — Supplementary Material 1 [file 13036_2025_565_MOESM1_ESM.docx]

[Supplementary Material](https://www.mdpi.com/1660-3397/16/2/73/htm#app1-marinedrugs-16-00073)s

Antimicrobial film from poly(butylene succinate) and cymophenol as a sustainable approach to food waste reduction: Antimicrobial properties and its effects on soil microorganism, brine shrimp (*Artemia salina*) and fresh strawberry

**1. Additional figures for antimicrobial activity of the PBS/cymophenaol films**
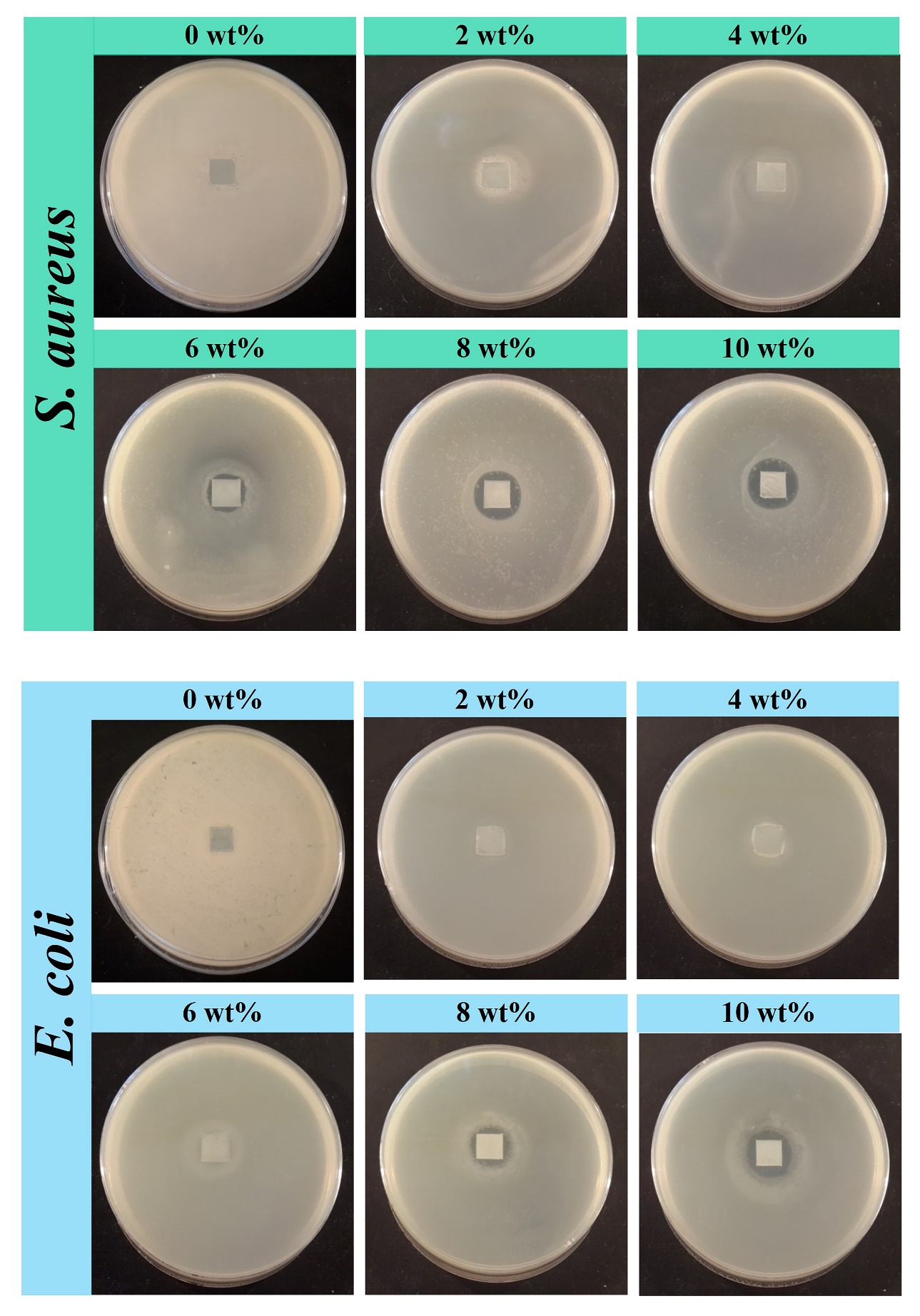


**Fig. S1** Antimicrobial activity against *S. aureus* and *E. Coli* of PBS/cymophenol films with 50 µm thickness at different cymophenol concentrations.


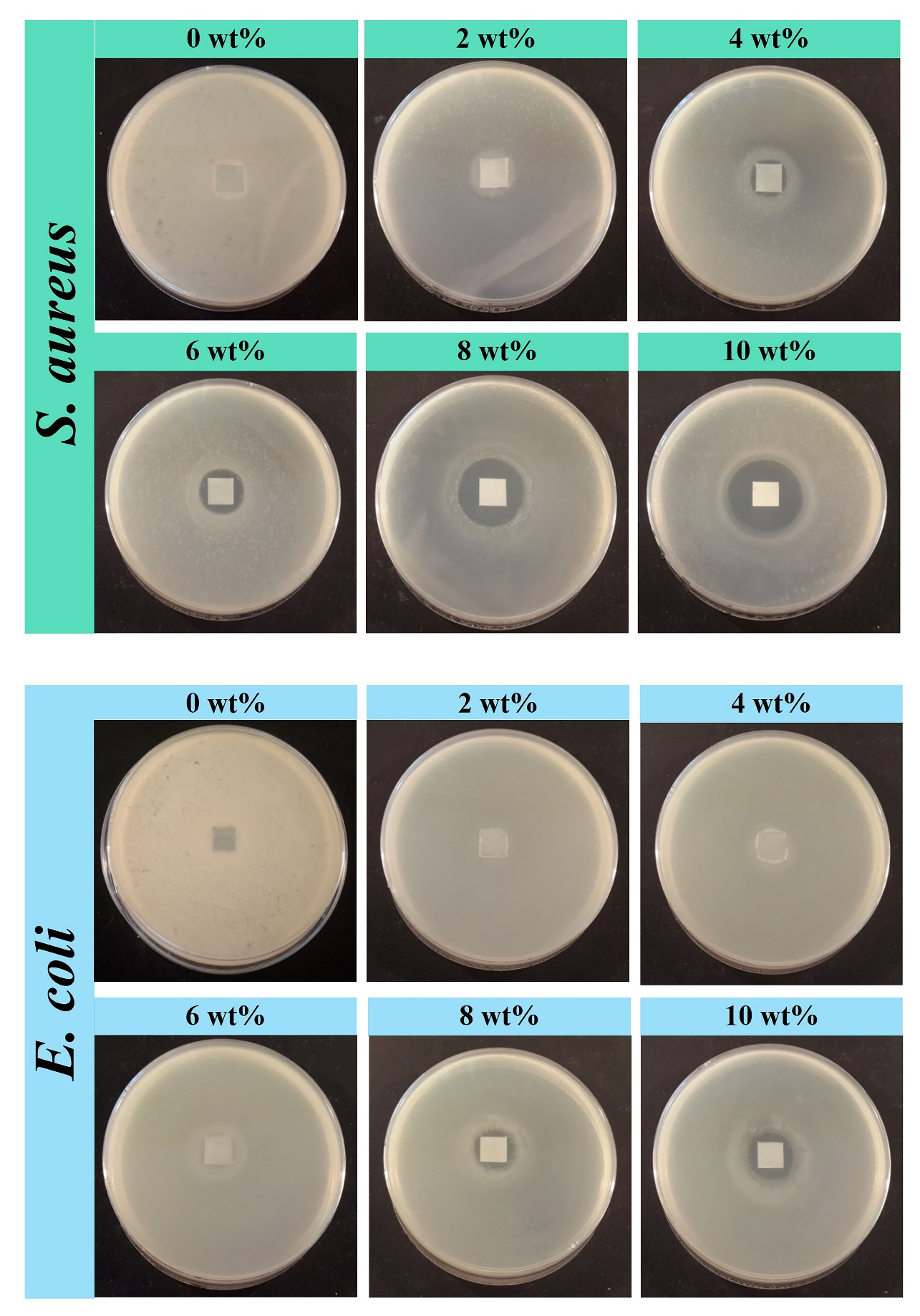


**Fig. S2** Antimicrobial activity against *S. aureus* and *E. Coli* of PBS/cymophenol films with 100 µm thickness at different cymophenol concentrations.
